# In the Abstract

**Published:** 1893-01-21

**Authors:** 


### PASSING TOPICS.

### IN THE ABSTRACT.

The interesting figures pourtraying the constituent contributions to the gross total collected by the Hospital Sunday Fund during the past twenty years which the Editor of the "Church of England Year-Book" has published, have furnished a text for highly congratulatory lucubrations by ecclesiastical and other journals which support the Establishment. We do not object to this; on the contrary, as a certain creation of Dickens was in the habit of remarking, we "only mention it." Nothing can be more natural or proper than that the Church should desire to take credit for what is without question singularly conspicuous evidence of her paramount social position. To paraphrase the well-known illustration, this is clearly a case of the Church first and the rest nowhere.

But after all, the inquiry we want seriously to make is, whether the Church can find anything, when the statistics of the Hospital Sunday Fund are carefully examined, about which she can be reasonably jubilant? It is not so much a question of how far the aggregate contributions of Churchgoers compare with those of any other religious body, or all others combined, as what is their duty to the sick poor, and how far does their assistance represent a due discharge of that duty when it is measured by the necessities of the case and their ability to meet them? Conclusions drawn from tables of statistics are, as we know, notoriously unreliable, and sets of figures considered abstractedly, when all their importance depends upon their relation to other figures, have only a misleading significance.

The maintenance of the Metropolitan Hospitals during the twenty years over which Canon Burnside's records are spread has cost, roundly stated, about fifteen millions sterling. Towards this total, the Sunday Fund collections have supplied something more than £600,000, and the Church something under half a million, or less than a thirtieth part of the whole expenditure. If we take the churchgoing population at a million, and divide the £478,000 among them, we arrive at the "less than sixpence a head" which we remember to have seen recorded somewhere as the average annual contribution of the congregational units.

It is no doubt the duty of the hospitals to be grateful for much smaller service than this, and that they do most sincerely, and without any *arrière pensée*, appreciate the annual grant they receive from the Sunday Fund, we have every reason to feel assured. But if the Church is to take the place in the affections of the people which the Archbishop of Canterbury, like all good Churchmen, desires it should occupy, we venture to think that the exaltation of Hospital Sunday to a high place among the festivals of the calendar is a necessity. For the Church to hold her own in this irreverent age, solicitude for spiritual welfare must be accompanied by evidence of a power and willingness to appraise and relieve physical distress and suffering. The more Christianity abjures mere abstract religion and identifies itself with the precepts and example of its Divine Founder, who "went about doing good," the more irrelevant will Mr. Robert Buchanan's somewhat shocking query become—"Is Christianity played out?" Agricola.

